# Accidental vs. Abusive Head Trauma in Infancy: Is Revival Shaking the Missing Link?

**DOI:** 10.1002/ccr3.9633

**Published:** 2024-11-29

**Authors:** Mark J. Greenwald

**Affiliations:** ^1^ Department of Ophthalmology and Visual Science University of Chicago Chicago Illinois USA

I wish to comment on the recent case report from Brook, Squier, and Mack [1], which describes an infant who was found to have bilateral subdural hemorrhage and extensive bilateral retinal hemorrhage (RH) after a short fall with occipital impact, which was captured on surveillance video. The authors attribute all of the hemorrhagic findings to the fall, which occurred less than 24 h prior to fundus examination and CT imaging. RHs were documented with a detailed description including drawings made by the examining ophthalmologist. The baby appeared to have made a full neurologic recovery within days of injury.

The ophthalmologist's description, to my reading of the handwritten note reproduced in of the report, includes the following observations: “… Large macular intraretinal (? preretinal) hemorrhage both eyes across macula. Almost all intraretinal dot blot + preretinal. Nil [illegible] flame hemorrhages seen. … (A) Lack of superficial flame hemorrhages indicates insult/injury likely to be ≥ 1 week ago. (B) Need to document resolution of hemorrhages to better confirm timing of injury.” It is further noted, with an arrow pointing to the structure in question (described in text as “a retinal fold along the inferior arcade of the right eye”), “? fold ? older schisis.”

In the text, it is subsequently stated “The IRHs resolved slowly, still present 1 week later,” followed by the comment “This indicates that the RHs occurred around the time of the fall and, given the changes to the state of the infant caused by the fall, it is reasonable to attribute the RHs to the fall.” While not directly contradicted by the facts as presented, I do not believe this conclusion is warranted based on the reported findings and their evolution, particularly given the history of a possible seizure weeks and a prior fall days before the documented incident, and head images that appear to be consistent with the chronic presence of at least a portion of the extra‐axial fluid collections they demonstrate. Acuteness of RH onset would in fact have been better supported by disappearance of most of them after 1 week, rather than the observed persistence [2]. The authors have not, in my opinion, adequately established either the very recent onset of extensive retinal hemorrhage, or the presence of a retinal fold typical of acute traumatic retinoschisis [3, 4].

A further concern, which relates more generally to the question of whether short falls can duplicate the clinicopathologic picture that is widely ascribed to abusive head trauma: We are given no information about what may have transpired immediately after the recorded incident. The video ends abruptly with the infant lying still on the floor. There is no indication of the immediate response of his caregivers. The mother “collected” him after an unspecified interval, finding him “floppy” and “unresponsive.” There was more than sufficient opportunity and reason to suspect the baby may have been subjected to “revival shaking” at the hands of one of the adults in whose care he remained until delivered to a hospital by his mother [5].

Since the earliest descriptions of shaken baby syndrome, the literature has been replete with histories of shaking done by a caregiver intent on helping a baby found unconscious or in acute distress [3, 6, 7]. A recent comprehensive review of confessions in presumed shaking injury cases confirmed that the second most commonly described scenario was attempted revival or relief from distress (31%, vs. incessant crying in 60%) [8]. Despite their pervasiveness and persistence, such narratives have, especially in more recent years, tended to be dismissed as fabricated or irrelevant [9]. There is, however, no sound empirical basis for this view.

All available evidence is in fact consistent with the possibility that a nearby adult can, with only good intentions, administer a succession of brisk shakes to an infant who has been momentarily incapacitated (by a blow to the head, choking, seizure, apneic episode, or other ALTE‐type event), which will cause the “triad” associated with many cases of abusive head trauma: retinal hemorrhage, intracranial hemorrhage, and encephalopathy (now regarded by some as a consequence of anoxic brain damage caused by apnea secondary to less‐than‐extreme kinking or stretching of the lower brainstem or cervical cord/nerve roots) [10, 11].

Furthermore, the possibility cannot be discounted that brief shaking, in a moment of panic or confusion, may fail to be reported or even recalled (as an action distinct from inexpertly performed CPR) by the perpetrator or eyewitnesses (Caffey, in his seminal 1972 paper, described “traumatic amnesia” on the part of those involved toward such impulsive and perhaps instinctual behavior [12].). This could truly be the “missing link” that has long blurred the boundary between accidental and inflicted head injuries in infancy.

It would be of enormous importance if there were additional video in this case that shows a caregiver shaking the baby in an attempt at revival. Presuming such is not the case, I believe that for this or any short fall in infancy, or other apparently minor head trauma, to be conclusively identified as the cause of the AHT‐linked triad (or two of its components), there must be continuous video documentation from the moment of injury until transfer to the care of EMS or other professional support, to exclude the possibility that the infant was shaken in the course of innocent but inappropriate resuscitation efforts.

The authors' contribution to the knowledge foundation of infant head trauma is welcome, but their work as presented does little to clarify the pathogenesis of ocular or cerebral hemorrhage in victims.

## Author Contributions

**Mark J. Greenwald:** conceptualization, writing – original draft, writing – review and editing.

## Data Availability

Data sharing not applicable to this article as no datasets were generated or analysed during the current study.
